# The extracellular HDAC6 ZnF UBP domain modulates the actin network and post-translational modifications of Tau

**DOI:** 10.1186/s12964-021-00736-9

**Published:** 2021-05-01

**Authors:** Abhishek Ankur Balmik, Shweta Kishor Sonawane, Subashchandrabose Chinnathambi

**Affiliations:** 1 Neurobiology Group, Division of Biochemical Sciences, CSIR-National Chemical Laboratory (CSIR-NCL), Dr. Homi Bhabha Road, Pune, 411008 India; 2 Academy of Scientific and Innovative Research (AcSIR), Ghaziabad, 201002 India

**Keywords:** HDAC6, Tau, Phosphorylation, Tauopathies, Microtubule, Actin, Podosomes, Podonut, Actin, Cytoskeleton, Neurodegeneration

## Abstract

**Background:**

Microtubule-associated protein Tau undergoes aggregation in Alzheimer`s disease (AD) and a group of other related diseases collectively known as Tauopathies. In AD, Tau forms aggregates, which are deposited intracellularly as neurofibrillary tangles. Histone deacetylase-6 (HDAC6) plays an important role in aggresome formation, where it recruits polyubiquitinated aggregates to the motor protein dynein.

**Methods:**

Here, we have studied the effects of HDAC6 ZnF UBP on Tau phosphorylation, ApoE localization, GSK-3β regulation and cytoskeletal organization in neuronal cells by immunocytochemical analysis. This analysis reveals that the cell exposure to the UBP-type zinc finger domain of HDAC6 (HDAC6 ZnF UBP) can modulate Tau phosphorylation and actin cytoskeleton organization.

**Results:**

HDAC6 ZnF UBP treatment to cells did not affect their viability and resulted in enhanced neurite extension and formation of structures similar to podosomes, lamellipodia and podonuts suggesting the role of this domain in actin re-organization. Also, HDAC6 ZnF UBP treatment caused increase in nuclear localization of ApoE and tubulin localization in microtubule organizing centre (MTOC). Therefore, our studies suggest the regulatory role of this domain in different aspects of neurodegenerative diseases. Upon HDAC6 ZnF UBP treatment, inactive phosphorylated form of GSK-3β increases without any change in total GSK-3β level.

**Conclusions:**

HDAC6 ZnF UBP was found to be involved in cytoskeletal re-organization by modulating actin dynamics and tubulin localization. Overall, our study suggests that ZnF domain of HDAC6 performs various regulatory functions apart from its classical function in aggresome formation in protein misfolding diseases.

**Video abstract**

**Supplementary Information:**

The online version contains supplementary material available at 10.1186/s12964-021-00736-9.

## Background

The misfolded proteins are either refolded by chaperones or targeted for degradation via ubiquitin proteasomal system (UPS) [1–4]. Upon failure or disruption of the UPS, aggregates are directed towards the formation of aggresomes, which serve as a cytoprotective response upon UPS failure [4–7]. HDAC6 is a class II histone deacetylase primarily present in cytoplasm. This protein is involved in regulation of various cellular functions. It consists of two catalytic deacetylase domains (histone deacetylase 1 and 2, residues 87–404 and 482–800 respectively) and a unique ZnF UBP (Zinc finger ubiquitin binding protein) domain (residues 1131–1192), which sets HDAC6 apart from other HDACs [8–10]. One of the major functions of HDAC6 is recruitment of polyubiquitinated protein aggregates to Dynein/Dynactin complex to sequester them to microtubule organizing centre (MTOC) in the perinuclear region for aggresome formation, thereby facilitating their clearance by autophagy [8–10]. Another important function of HDAC6 is the promotion of autophagosome-lysosome fusion and completion of autophagy [11–13]. The function of HDAC6 in both UPS and autophagy indicate its role as a possible link between the two mechanisms [11–13]. The impairment of UPS function acts as a cue for the activation of compensatory mechanisms for clearance of protein aggregates. It has been studied in the *Drosophilla* model of spinobulbar muscular atrophy, where expression of HDAC6 has been found to effectively cause rescue from UPS impairment-induced neurodegeneration by triggering the autophagic clearance of protein aggregates [12]. In another study, HeLa cells transfected with PolyQ Huntingtin showed the formation of intracellular protein aggregates, which require HDAC6 for autophagic clearance after inhibition of proteasomal system [14]. Overall, the function of HDAC6 with respect to protein aggregate clearance and autophagy induction serves as an important protective mechanism. In line with these considerations, expression levels of HDAC6 increases sharply in protein misfolding diseases [15]. The functions of ZnF UBP domain of HDAC6 are less understood as compared to the function of its catalytic domains. The possible role of HDAC6 ZnF UBP in directly modulating the aggregation propensity and stability of Tau has been recently reported [16].

Phosphorylation is a well-studied PTM of Tau responsible for its conversion to pathological form during AD pathogenesis [17–19]. Alzheimer`s disease is associated with upregulation of cellular kinases, such as GSK-3β and CDK5 [20]. GSK-3β is one of the major kinases involved in Tauopathies [21]. Tau is phosphorylated by GSK-3β on both primed (after pre-phosphorylation) and unprimed sites and such phosphorylation affects the ability of Tau to bind and stabilize microtubules [22, 23]. Many serine and threonine residues within the repeat region of Tau have been mapped as potential phosphorylation sites by proline-directed kinases [24]. On the other hand, protein phosphatases, such as PP1 and PP2A, are known to be downregulated in AD, failing to reverse the effect of hyperphosphorylation [25–27]. Therefore, there is an imbalance between the kinase and phosphatase function in neurons under pre-pathological conditions. We have studied the effects of HDAC6 ZnF UBP domain on phospho-Tau epitopes—pT181 and AT8, as well as on the level of total and downregulated form of GSK-3β. HDAC6 ZnF UBP was found to lower both phospho-epitopes and promoted GSK-3β downregulation.

Another important aspect of neurodegenerative diseases involve mis-functioning of cytoskeletal elements. Microtubule network and actin organization were found to be distorted in neurodegeneration leading to impaired cellular trafficking and distortion of other associated functions [28, 29]. Actin organization is crucial for synaptic signalling in neurons, where it is involved in formation of dendritic spines for neurotransmission. Impaired actin assembly and depolymerisation lead to the loss of dendritic spines, ultimately causing neuronal death [30, 31]. HDAC6 is a key protein in the regulation of both actin and microtubule organization through its deacetylase activity [32]. HDAC6 acts on cortactin and mediates its association with F-actin (fibrous actin) to facilitate cell motility [33]. In present study, we studied the role of HDAC6 ZnF UBP domain in modulation of cytoskeletal assembly independent of its catalytic domains. The localization of ApoE either in cytoplasm or nucleus serves as an indicator of neuronal health. Nuclear localization of ApoE is associated with increased neuronal survival. Enhanced ApoE nuclear localization was observed in neurons in the presence of HDAC6 ZnF UBP, suggesting that HDAC6 ZnF UBP can promote ApoE nuclear localization in HSP90 and nucleolin mediated manner.

Overall, in this study, HDAC6 ZnF UBP domain was found to affect actin and tubulin organization, Tau phosphorylation, and localization of ApoE in neuronal cells. Enhanced formation of podosome and lamellipodia-like structures was found, when neuronal cells were exposed to HDAC6 ZnF UBP domain, suggesting direct role of this domain in actin organization. HDAC6 ZnF UBP treatment also resulted in enhanced tubulin localization in MTOC, indicating the possible role of this domain in tubulin polymerization events as well. Our findings suggest that HDAC6 ZnF UBP acts as a direct modulator of Tau phosphorylation and cytoskeletal dynamics.

### Materials and methods

#### Chemicals and reagents

Luria–Bertani broth (Himedia); Ampicillin, NaCl, Phenylmethylsulfonylfluoride (PMSF), MgCl_2,_ APS, DMSO, Ethanol (Mol Bio grade), Isopropanol (Mol Bio grade) were purchased from MP biomedicals; IPTG and Dithiothreitol (DTT) from Calbiochem; MES, BES, SDSfrom Sigma; EGTA, Protease inhibitor cocktail, Tris base, 40% Acrylamide, TEMED from Invitrogen. For cell culture studies, Dulbecco modified eagle’s media (DMEM), Fetal bovine Serum (FBS), Horse serum, Phosphate buffer saline (PBS, cell biology grade), Trypsin–EDTA, Penicillin–streptomycin, Pierce™ LDH Cytotoxicity Assay Kit (Thermo, cat no 88953), RIPA buffer were also purchased from Invitrogen. MTT reagent, Okadaic acid and TritonX-100 were purchased from Sigma. The coverslip of 18 mm was purchased from Bluestar for immunofluorescence. In immunofluorescence and western blot study we used the following antibodies: Beta-actin (Thermofisher cat no. MA515739) Beta Tubulin (BT7R) (Thermofisher, cat no MA516308) and total Tau antibody K9JA (Dako, cat no A0024), pT181 (Invitrogen, cat no 701530) AT8 (Thermo fisher, cat no MN1020), GSK-3β (Thermo fisher, cat no MA5-15109), Phospho-GSK-3β (Ser9) (Thermo fisher, cat no MA5-14873), anti-ApoE (Sigma, cat no. SAB2701946), anti-mouse secondary antibody conjugated with Alexa Fluor-488 (Invitrogen, cat no A-11001), Goat anti-Rabbit IgG (H + L) Cross-Adsorbed Secondary Antibody with Alexa Fluor 555 (A-21428), Rabbit anti-Goat IgG (H + L) Cross-Adsorbed Secondary Antibody with Alexa Fluor 594 (A27016) and DAPI (Invitrogen).

#### Protein expression and purification

Full length Tau (hTau40wt) in pT7C were transformed and expressed in BL21* cells while HDAC6 ZnF UBP in pET28a-LIC was transformed and expressed in BL21 Codon plus RIL cells. Full-length Tau and repeat domain Tau were purified in two steps using cation exchange chromatography and size-exclusion chromatography. The cells expressing these proteins after transformation were scaled up and harvested. The cells were lysed by homogenization at 15,000 KPSI. The lysate was supplied with 0.5 M NaCl and 5 mM DTT and kept at 90 °C for 20 min to denature all structured protein. The resulting sample was centrifuged at 40,000 rpm for 45 min. The supernatant was kept for overnight dialysis in 20 mM MES pH 6.8. The dialyzed sample was centrifuged again at 40,000 rpm for 45 min and the supernatant was filtered and loaded onto Sepharose fast flow (SPFF) column pre-equilibrated with 20 mM MES pH 6.8, 50 mM NaCl. Elution was carried out using 20 mM MES pH 6.8, 1 M NaCl. The fractions collected from cation exchange chromatography containing Tau protein were pooled, concentrated and subjected to size-exclusion chromatography using 1X PBS, 2 mM DTT in Superdex 75 Hi-load 16/600 column [34, 35]. Purification of HDAC6 ZnF UBP was carried out by Ni–NTA affinity chromatography using 50 mM Tris–Cl pH 8.0 with 20 mM Imidazole for wash and 1000 mM imidazole for elution. The sample was dialyzed overnight in 50 mM Tris–Cl pH 8.0, 100 mM NaCl, 2.5% glycerol to remove imidazole followed by size-exclusion chromatography using Superdex 75 Hi-load 16/600 column [36].

#### Cell viability by MTT assay

The effective concentration of HDAC6 for subsequent treatments was determined by studying the concentration dependent toxicity studies by MTT assay. 10^4^ neuro2a cells (ATCC CCL-131) were seeded in a 96 well culture plate in DMEM supplemented with 10% FBS and antibiotic penicillin–streptomycin for 24 h at 37 °C CO_2_ incubator. The cells were treated with HDAC6 (0–500 nM) in serum-starved media for 24 h. MTT at a concentration of 0.5 mg/mL was added to the cells and incubated for 3 h. The reduction of MTT by cellular enzymes forms formazon crystals, which were dissolved in DMSO, and the colour developed was quantified by reading at 570 nm in a TECAN Infinite 200 PRO plate reader.

#### LDH assay

The effect of  HDAC6 0-500 nM treatment on cell membrane integrity was studied by LDH (Lactate Dehydrogenase) assay. The disruption of cell membrane integrity leads to the leakage of LDH enzyme, which is quantified by an enzymatic reaction giving a colored end product. For performing LDH assay, the cells were incubated and treated as mentioned for MTT assay. After treatment with HDAC6, supernatant media was used and assay was performed according to manufacturer’s protocol. In brief, 50 µL of cell supernatant was incubated with 50 µL of the reaction mixture provided for 30 min at room temperature. 50 µL of stop solution was added to each well and the colour developed was measured at 490 nm and background subtraction at 680 nm was done.

#### Caspase 3/7 activity assay

In order to study the effect of HDAC6 on inducing apoptotic cell death, activity of executioner caspase 3 was determined by EnzChek™ Caspase-3 Assay Kit. 10,000 cells/well cells were seeded in a 12 well culture plate for 24 h and further treated with HDAC6 (0–500 nM) for 24 h in serum-starved media. Caspase activity was performed as per manufacturer’s protocol. The cells were lysed with provided lysis buffer in freeze–thaw cycles. The cell debris was centrifuged out and supernatant was incubated with fluorescent substrate (DEVD-Rhodamine). The fluorescence was quantified at (Ex/Em) 496 /520 nm at different time intervals in TECAN Infinite 200 PRO plate reader.

#### Cell culture, immunofluorescence and quantitative analysis

Neuro2a cells from passage number 10–20 were cultured in advanced DMEM supplemented with penstrep-glutamine, anti-mycotic and 10% FBS. For immunofluorescence studies 5X10^4^ cells were seeded on a glass coverslip (Bluestar) in a 12 well culture plate. Cells were given desired treatment in serum starved media (0.5% FBS) for 24 h including groups involving 25 nM Okadaic acid (OA) treatment. After incubation period, cells were washed with PBS and fixed with 4% paraformaldehyde. Further cells were washed with PBS thrice and permiabilized using 0.2% Triton X-100. Cells were blocked with 2% horse serum and incubated with primary antibodies in a moist chamber at 4 ºC overnight. Next day, cells were washed thrice with 1X PBS and incubated with alexa fluor labeled secondary antibodies for 1 h at 37 ºC. The unbound secondary antibody was washed off with three washes of PBS and counterstained with DAPI. The coverslips were mounted in 80% glycerol and observed under 63X oil immersion lens in Axio Observer 7.0 Apotome 2.0 (Zeiss) microscope. The quantification of immunofluorescence intensity was carried out by Zen 2.3 software and mean fluorescence intensity per unit area were determined for various treatment groups in respective number of fields (n).

#### Statistical analysis

Two-tailed unpaired student t-test was used to determine the significance for experiments involving comparison of two groups (n.s.—non-significant, * indicates *P* ≤ 0.05, ** indicates *P* ≤ 0.01, *** indicates *P* ≤ 0.001). One way ANOVA was conducted for the levels of phospho-epitopes (pT181 and AT8) to compare different treatment groups. Tukey’s HSD (Honest significant difference) test was performed to compare the significance within groups (Significant at Tukey’s HSD *p* < 0.05). All the experiments were performed in triplicates and analyzed by Sigmaplot 10.0 (Systat software).

## Results

### HDAC6 ZnF UBP is non-toxic and non-apoptotic

Neuronal cells were exposed to HDAC6 ZnF UBP exogenously and effects of this exposure on different cellular functions were analyzed (Additional file 1: Fig. 1A). The cell viability analysis carried out by MTT assay showed no noticeable decrease in viability, which was maintained at 80% even in the presence of highest HDAC6 ZnF UBP concentration of 500 nM (Additional file 1: Fig. 1B). Similarly, LDH assay was utilized to check the effect of HDAC6 on membrane integrity in terms of LDH release. Neuronal cells showed intact membrane when treated with HDAC6 ZnF UBP in 20–500 nM concentration range (Additional file 1: Fig. 1C). Neuronal cells were treated with a range of concentrations of HDAC6 ZnF UBP (20–500 nM), and its internalization was monitored by immunostaining for HDAC6 and anti-His-tag (Additional file 1: Fig. 2). To further confirm the non-toxic nature of HDAC6 ZnF UBP for neuronal cells, we used caspase-3 assay to study the induction of apoptosis (if any) associated with treatment of cells with HDAC6 ZnF UBP. Under stressful conditions, cells undergo apoptosis mediated by endoproteases called caspases [37, 38]. Caspase-3 is an executioner caspase responsible for DNA fragmentation and degradation of cytoplasmic proteins [39–41]. The caspase-3 assay showed basal level of activity in all the treated and control samples. No difference was observed between HDAC6 ZnF UBP treated and untreated control samples in terms of cell viability, suggesting that HDAC6 ZnF UBP treatment did not induce apoptosis in neuro2a cells (Additional file 1: Fig. 1D). Therefore, these studies on cell viability and morphology inferred that HDAC6 ZnF UBP treatment did not show cytotoxic effects on neuro2a cells. Hence, in subsequent experiments, cells were treated with a moderate concentration of 50 nM HDAC6 ZnF UBP, unless stated otherwise.

**Fig. 1:**
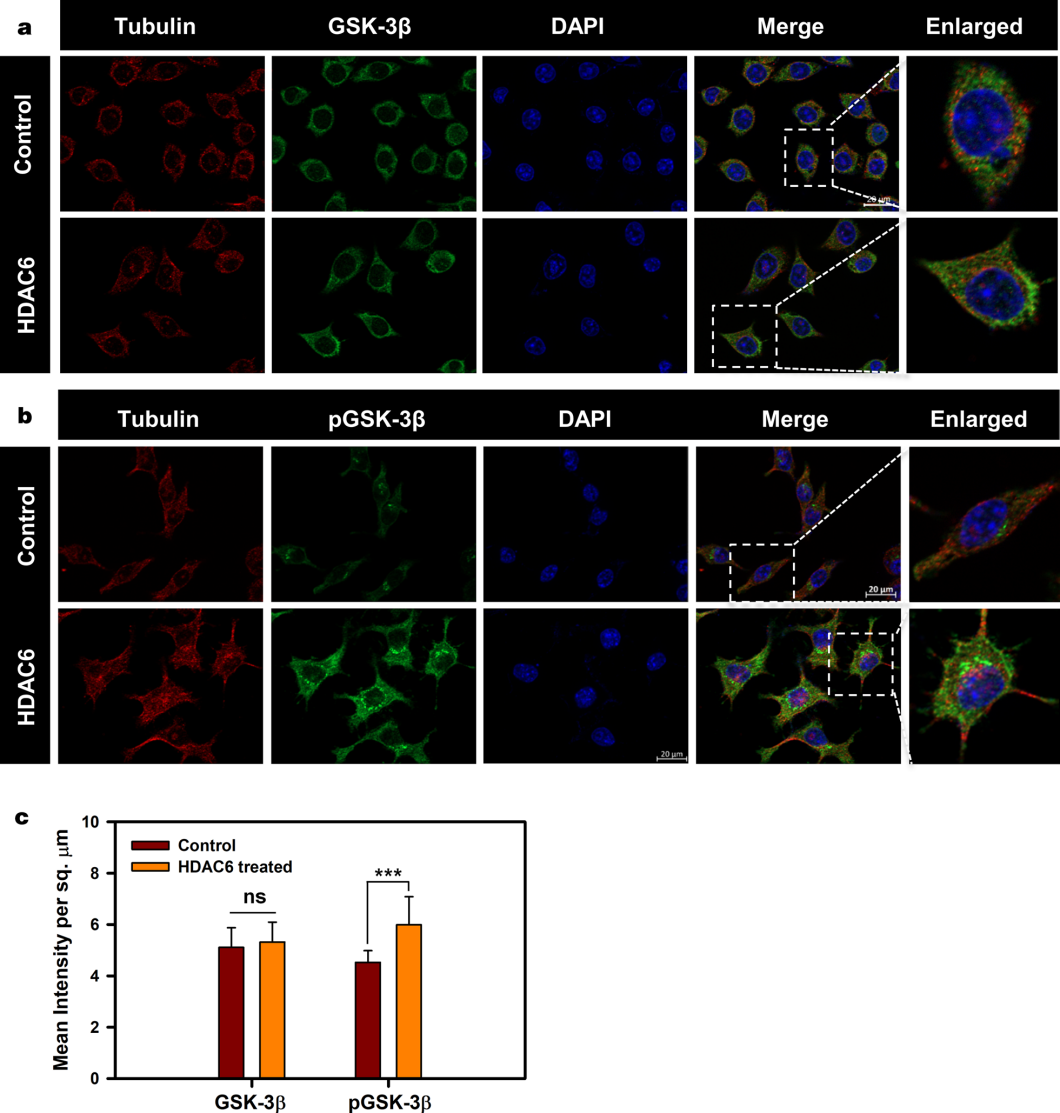
Downregulation of GSK-3β activity by HDAC6. **a** Neuro2a mapped for total GSK-3β shows their unaltered levels upon HDAC6 ZnF UBP. **b** Inhibitory phosphorylation of GSK-3β at Ser9 increases upon HDAC6 ZnF UBP treatment. The enlarged image shows the elevated level of pGSK-3β compared to neuro2a cell control. **c** Quantification of mean fluorescence intensity for GSK-3β in cell control and HDAC6 ZnF UBP treated cells showed non-significant difference while the levels of pGSK-3β were significantly increased upon HDAC6 ZnF UBP treatment in comparison to cell control

**Fig. 2:**
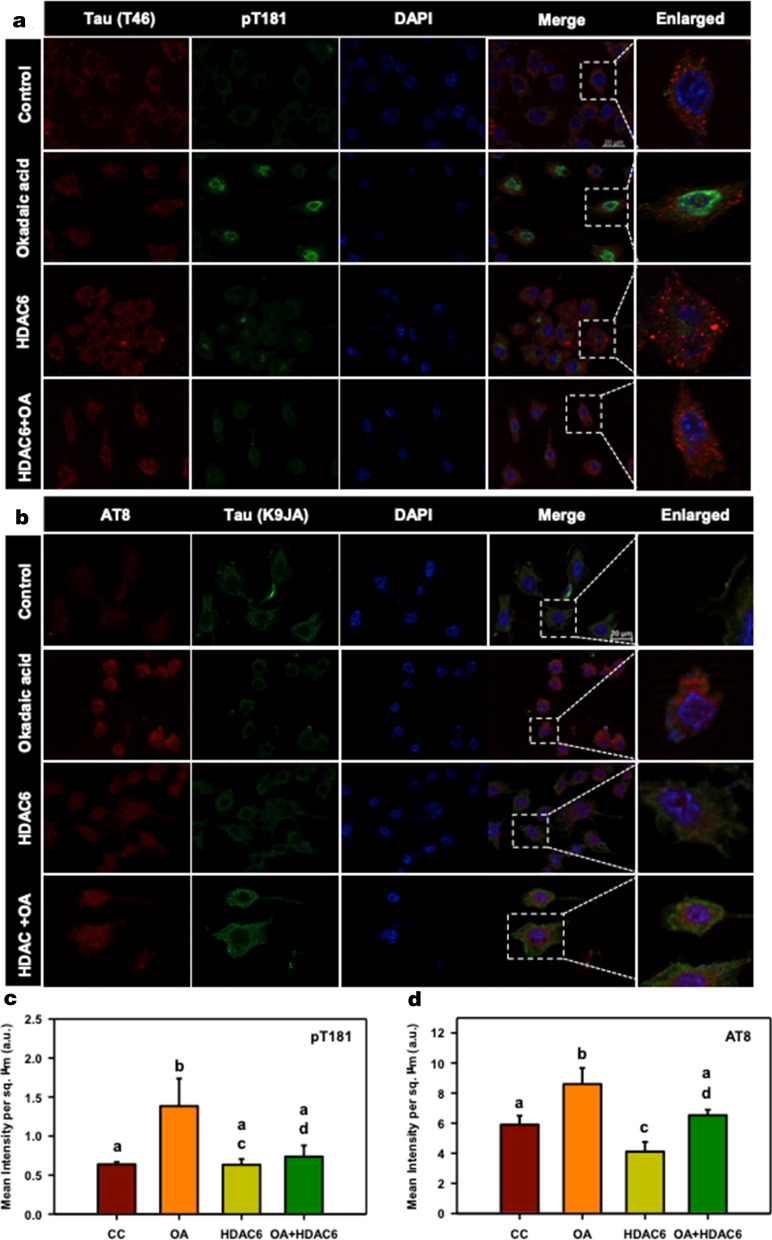
Inhibition of Tau phosphorylation by HDAC6. **a** HDAC6 treatment for 24 h along with OA shows inhibition of Tau phosphorylation at pT181 epitope whereas OA treatment shows increased phosphorylation at pT181. **b** Phosphorylation at AT8 epitope is also reduced in presence of HDAC and OA as compared to OA alone. The enlarged image of neuro2a treated with OA alone and OA along with HDAC6 ZnF UBP shows marked difference in the level of phosphorylation at pT181 and AT8 epitopes. **c**, **d** Mean fluorescence intensity for AT8 immunostaining of untreated neuro2a (CC), okadaic acid treatment (OA), HDAC6 ZnF UBP treatment (HDAC6) and HDAC6 ZnF UBP treatment along with okadaic acid. Okadaic acid treatment resulted in increased level of pT181 and AT8 phospho-epitopes. HDAC6 ZnF UBP treatment with OA reduced the level of both phospho-epitopes. Quantification data was analyzed by one way ANOVA followed by Tukey’s HSD test. Significant at mean difference > T (Tukey’s criterion) and α = 0.05. Groups with the same letters are not significantly different. The value of T is calculated as 0.365 for pT181 and 1.341 for AT8 quantification

### HDAC6 ZnF UBP enhances pGSK-3β levels

We mapped GSK-3β and pGSK-3β (pSer9) levels by immunofluorescence in neuronal cells in basal conditions and upon HDAC6 ZnF UBP treatment (No. of fields selected for quantification of GSK-3β and pGSK-3β (pSer9), n = 6). There was no marked difference in GSK-3β levels in HDAC6-treated and untreated cells (Fig. 1a). However, immunofluorescence analysis of HDAC6 ZnF UBP-treated cells showed significant increase in pGSK-3β levels (Fig. 1b). GSK-3β function in cells is regulated mainly by the inhibitory phosphorylation at Ser9 or Ser21 residues. The kinase activity of GSK-3β is reduced with phosphorylation at Ser9, as it affects the binding of primed substrates to GSK-3β. Mean fluorescence intensity of cell control and HDAC6 ZnF UBP treated cells showed non-significant changes in GSK-3β levels but pGSK-3β levels were found to be increased in HDAC6 ZnF UBP treated cells (Fig. 1c). The increase in pGSK-3β in neuronal cells signifies reduced GSK-3β activity on primed Tau substrate [42]. The HDAC6 ZnF UBP treatment of cells resulted in the increased levels of pGSK-3β (Ser9), which in turn enables reduced Tau phosphorylation. The increased levels of pGSK-3β suggested down-regulated GSK-3β activity under the influence of HDAC6 ZnF UBP.

### HDAC6 ZnF UBP reduces Tau phosphorylation in neuronal cells

Aberrant Tau phosphorylation is one of the key events in AD pathogenesis. Tau phosphorylation is required for its function in microtubule interaction and stabilization. Under pathological conditions, Tau becomes hyperphosphorylated due to imbalance in kinase and phosphatase levels or activity [17–19, 43] Okadaic acid (OA) was used as an inducer of hyperphosphorylation, as it inhibits protein phosphatase 2A (PP2A), thereby increasing the overall phosphorylation level [44]. Untreated control cells and HDAC6 ZnF UBP-treated cells alone showed basal levels of phospho-Tau at epitopes pT181 and pS202/T205 (AT8). The levels of pT181 were increased in OA treated cells. Cells supplemented with HDAC6 ZnF UBP with OA showed lower levels of phospho Tau at T181 as compared to the positive control (Fig. 2a). Tau phosphorylated at pT181 was dominantly seen in the nucleus, especially in positive control (enlarged images). Similar results were observed for phospho Tau epitope AT8. The enlarged images clearly show increased levels of phospho-AT8 in positive control as compared to OA + HDAC6 ZnF UBP treatment (Fig. 2b). pT181 and AT8 are two of the crucial epitopes of pathological Tau in AD. HDAC6 ZnF UBP treatment lowered down the level of these two phospho-epitopes, suggesting the possible role of this domain in modulating Tau phosphorylation. The level of both phospho-tau epitopes (pT181 and AT8) were quantified by their immunofluorescence levels in different experimental groups (No. of fields selected for quantification of pT181 and AT8, n = 5). Untreated cells (CC) showed no significant difference from HDAC6-treated or OA + HDAC6-treated cells in the levels of pT181and AT8 immunostaining. OA treatment resulted in increased pT181and AT8 levels, which were found to be reduced with HDAC6 ZnF UBP treatment (Fig. 2c, d). Quantification data were analyzed by one-way ANOVA followed by Tukey’s HSD test (significant at α = 0.05). The statistical significance was calculated with respect to Tukey’s criterion (T). The groups with same letters show no significant difference.

### HDAC6 ZnF UBP modulates actin dynamics

**Fig. 3:**
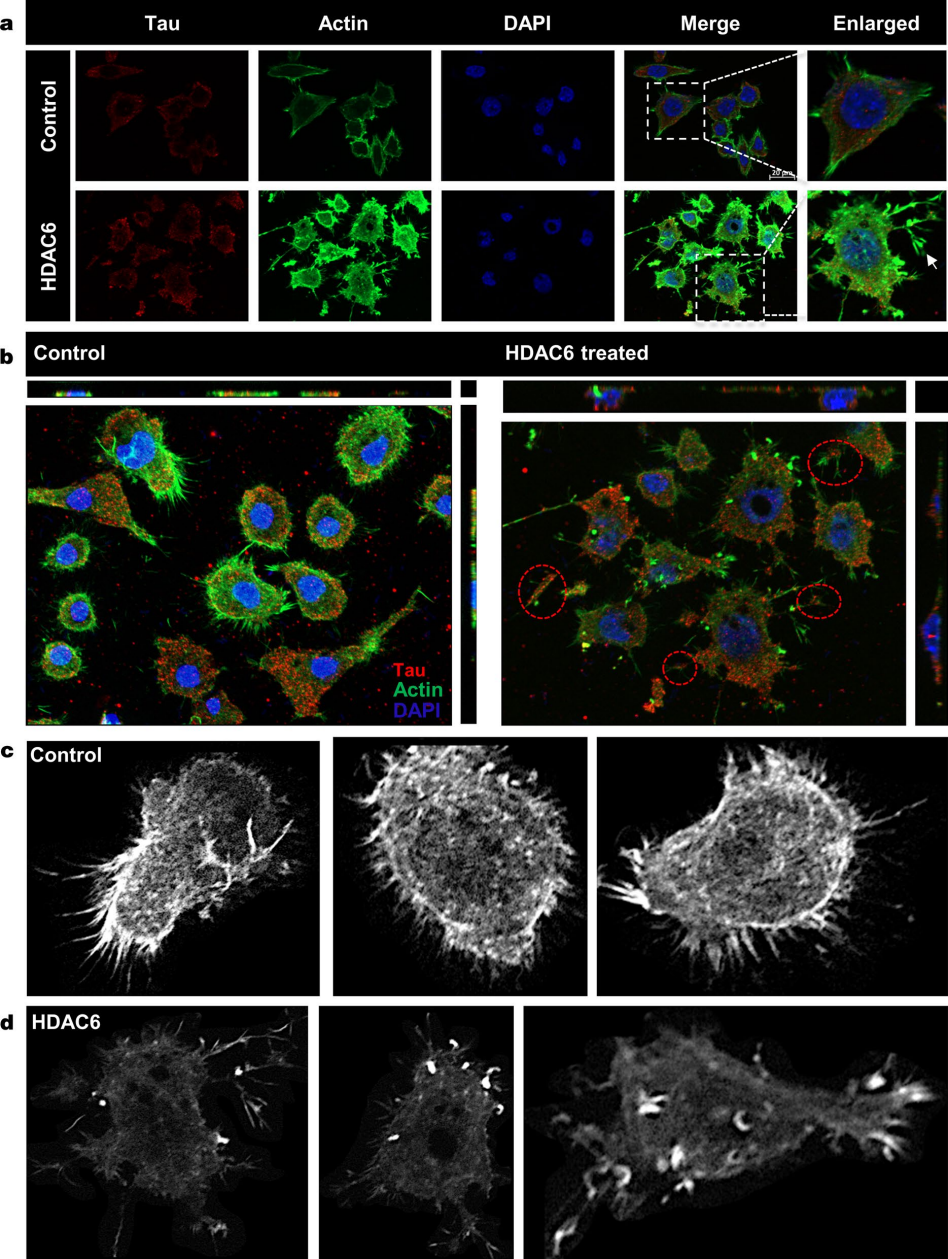
HDAC6 as a modulator of actin dynamics. HDAC6 deacetylase activity is known to modulate actin dynamics. The effect on actin dynamics was studied after exposure to HDAC6 ZnF UBP domain. HDAC6 ZnF UBP treatment to neuro2a resulted in enhancement of neurite extensions which are mapped by F-actin staining. **a** HDAC6 ZnF UBP treatment for 24 h increases the neuronal extensions as compared to untreated control cells when the extensions are mapped by actin immunostaining. The Tau levels remain unaltered in both the groups. **b** The orthogonal projection shows increase in the number of neurites in HDAC6 ZnF UBP treated cells. **c** Greyscale images for untreated neuro2a cells mapped for F-actin showed small extensions in the growth cones. **d** In HDAC6 ZnF UBP treated cells, F-actin containing longer extensions were observed. Growth cones were observed to be concentrated in membrane extensions and filopodia-like structures

In neuronal cells, actin dynamics is important for cell-to-cell communication through regulating neurite extension. HDAC6 is known to modulate actin organization through its association with and deacetylase activity on cortactin and arp2/3 complex [33, 45, 46]. The function of HDAC6 ZnF UBP domain in actin dynamics is not understood as of yet. Our observations showed that HDAC6 ZnF UBP treatment can lead to an increase in neuritic extensions in neuro2a cells. We mapped HDAC6 ZnF UBP-treated cells with FITC-Phalloidin to observe the changes in F-actin cytoskeleton. We studied actin organization along with Tau and observed that HDAC6 ZnF UBP treatment had no effect on Tau levels but the actin cytoskeleton changed significantly (Fig. 3a). Further, to check for the changes in Tau localization on treatment, we mapped actin with Tau. The control cells showed a normal F-actin cytoskeleton in the extensions, but HDAC6-treated cells showed presence of actin along with Tau in these extensions (Fig. 3b). We also observed Tau-actin co-localization in neuritic extensions indicating the interplay of microtubule and actin dynamics in formation of cell extensions (Additional file 1: Fig. S3A, B). Greyscale images for actin immunostaining shows localization in neurite extensions and growth cones in filopodia-like structures (Fig. 3c, d).

### Enhancement of podosome-like structures by HDAC6 ZnF UBP

**Fig. 4:**
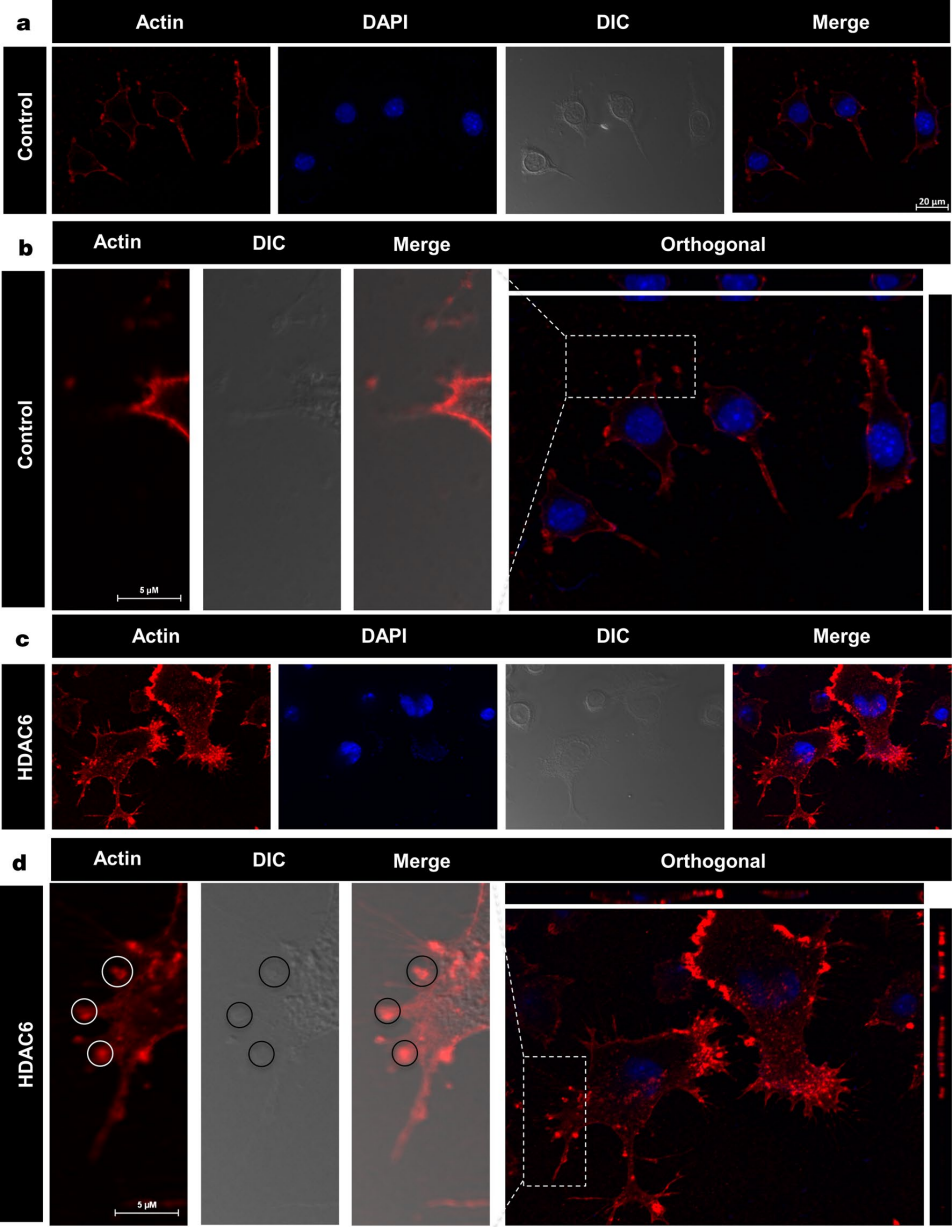
Enhancement of podosome formation by HDAC6. **a** Podosomes marked by actin rich structures along plasma membrane, were observed in neuro2a cells at a minimal level. **b** Enhancement in podosome like structures observed in HDAC6 ZnF UBP treated cells. Orthogonal projection images showed marked difference in membrane morphology and actin concentrated in membrane ruffles and podosome like structures in HDAC6 ZnF UBP treated cells. **c** HDAC6 is found to be present in neuritic extensions along with actin suggesting its role in regulation of neurite extensions. This is not seen in case of untreated cells as indicated in enlarged images for both groups. **d** In HDAC6 ZnF UBP treated cells, F-actin containing longer extensions were observed. Growth cones were observed to be concentrated in membrane extensions and filopodia-like structures

**Fig. 5:**
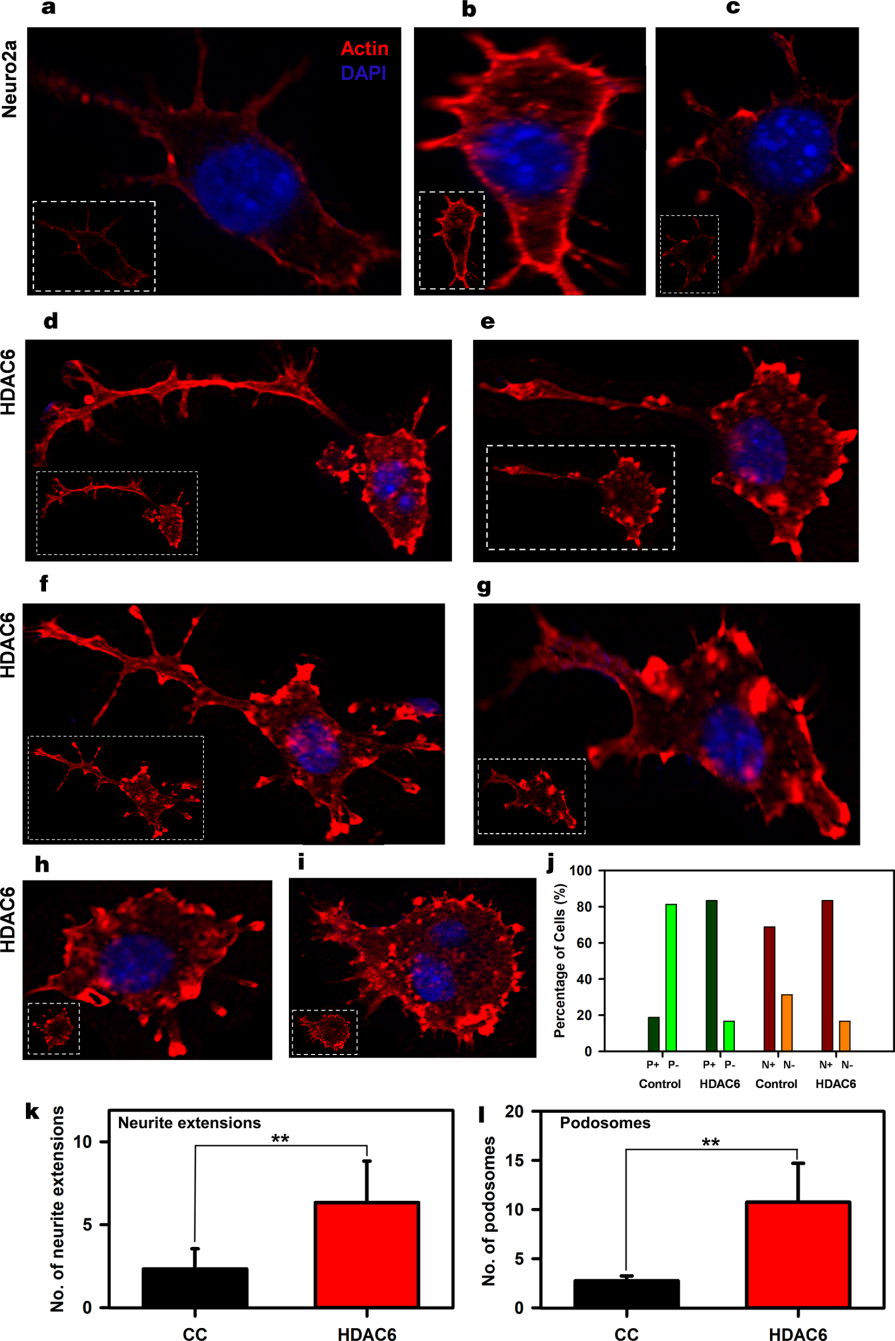
Podosome, lamellipodia and podonut-like structure induced by HDAC6 ZnF UBP. Neuro2a cells exposed to HDAC6 ZnF UBP exhibit a variety of actin rich structures characteristics of migratory or invading cells. **a**–**c**) Untreated neuro2a cells showed actin uniformly distributed along the periphery with small neuritic extensions while **d**, **e** HDAC6 ZnF UBP treated cells showed longer extensions and membranes ruffles resembling podosomes involved in cell migration. **f**, **g** Invadopodia and lamellipodia-like structures (encircled) were also observed, which are found in phagocytic cells. **h**, **i** Most of the treated cells were observed to consist of assemblance of podosomes (encircled) and podosome clusters called podonuts (white arrow) rich in actin. **j** The percentage of cells with or without podosomes/neurite extensions was calculated for control and HDAC6 ZnF UBP treatments groups. There were < 20% cells in control with podosome-like structures while > 80% cells in exhibited podosome-like structures upon HDAC6 ZnF UBP treatments. In cell control, 65–70% cells had neurite extensions, which were calculated to be 80–85% in HDAC6 ZnF UBP treated cells. (P + and P- represent cells with and without podosomes respectively. N + and N- represent cells with or without neuritic extensions respectively.) **k** The overall neurite extensions in neuro2a cells were counted in untreated and HDAC6 treated group in β-actin immunostained cells. The number of extensions in HDAC6 treated cells were found to be greatly enhanced compared to untreated group. **l** The podosomes formed after HDAC6 ZnF UBP treatment were quantified by counting the podosome crown structures in both untreated and treated groups. HDAC6 ZnF UBP treated cells showed more podosome clusters signifying its actin modulating effect. Statistical significance determined by two-tailed unpaired t-test. (n.s.—non-significant, * indicates *P* ≤ 0.05, ** indicates *P* ≤ 0.01, *** indicates *P* ≤ 0.001)

Morphological changes in neuronal cells were observed when treated with HDAC6 ZnF UBP (Additional file 1: Fig. 4). HDAC6 ZnF UBP-treated cells showed increased membrane ruffles and podosome-like structures. Neuro2a cells were incubated with 50 nM HDAC6 ZnF UBP on 18 mm coverslips for 24 h prior to immunostaining preparation. Immunostaining showed localization of actin in the membrane ruffles in HDAC6 ZnF UBP-treated cells. We observed minimal neuritic extensions in untreated neuro2a as compared to treated cells (Fig. 4a). Actin localization was observed mostly along the cell periphery in untreated neuro2a, while it was focused in the neurite extensions and protruding podosome-like structure in HDAC6 ZnF UBP-treated cells (Fig. 4b). Orthogonal sections were taken to clearly visualize F-actin driven extensions and podosome formation. The initial orthogonal sections showed numerous extensions, and actin localized in the tip of extensions in HDAC6 ZnF UBP-treated cells as compared to control (Fig. 4c, d). Involvement of podosomes in cell adhesion and migration is an important attribute of invasive cells. Neuro2a, being a neuroblastoma cell line, can form podosomes for cell migration and attachment. In contrast to untreated control neuronal cells (Fig. 5a–c), HDAC6 ZnF UBP-treated cells showed enhanced cell extensions and actin-rich structures similar to podosomes formed in migrating and invading cells. HDAC6-treated cells showed a variety of actin-based membrane protrusions which can be morphologically classified as filopodia or lamellipodia, podosomes and podonuts (Fig. 5d–i). The neuritic extensions and podosomes were quantified in untreated control cells (CC) and HDAC6-treated cells. The neurites and podosomes were counted manually in random multiple fields for untreated control and HDAC6 treated group (Total no. of fields, n = 6). Overall, < 20% podosomes containing cells were observed in cell control while > 80% of HDAC6 ZnF UBP treated cells exhibited podosomes-like structures. Similarly, 65–70% cells with neurite extensions were found in cell control while 80–85% cells had enhanced neurite extensions in HDAC6 treated cells (Fig. 5j). HDAC6 ZnF UBP treated cells showed significantly enhanced neurite extensions and podosomes compared to untreated cells implicating its effect on actin dynamics and re-organization (Fig. 5k, l). Statistical significance of control and HDAC6 treated cells was analyzed by two-tailed unpaired student t-test.

### HDAC6 ZnF UBP enhances ApoE nuclear localization

**Fig. 6:**
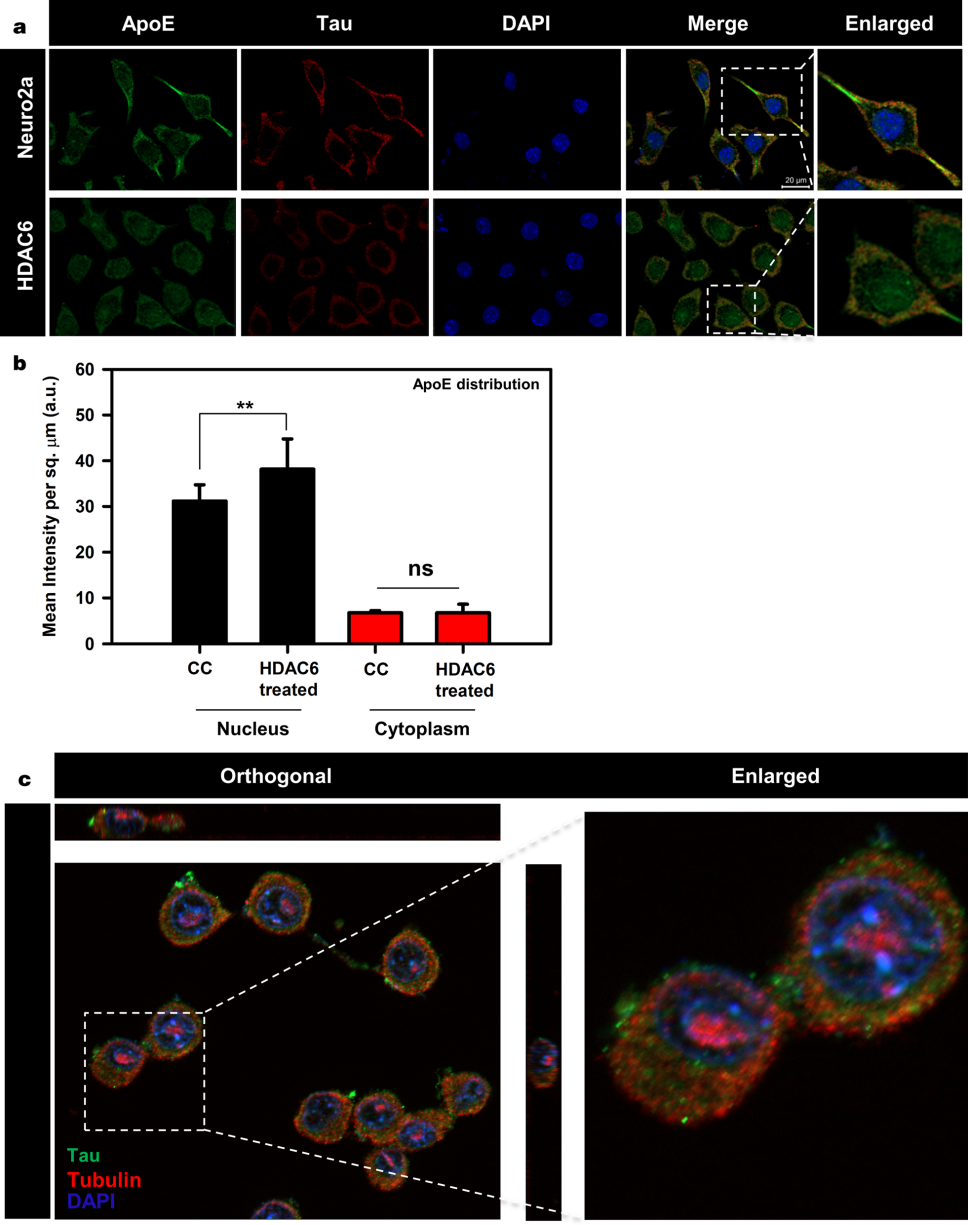
Modulation of ApoE and Tubulin localization by HDAC6. **a** Immunofluorescence mapping for ApoE and Tau upon HDAC6 treatment in neuro2a cells shows increased nuclear localization of ApoE. **b** ApoE intensity in nucleus and cytoplasm was quantified for untreated and HDAC6 ZnF UBP treated neuro2a immunostained with anti-ApoE antibody. There was no significant difference in cytoplasmic level of ApoE in both groups while nuclear ApoE fraction was notably increased in HDAC6 ZnF UBP treated cells. **c** Neuro2a treated with HDAC6 ZnF UBP shows localization of tubulin focused over nuclear periphery compared to untreated neuro2a where tubulin is evenly distributed in cytoplasm. Orthogonal projection image shows tubulin localization in nuclear periphery in HDAC6 ZnF UBP treated neuro2a cells. Statistical significance determined by two-tailed unpaired t-test. (n.s.—non-significant, * indicates *P* ≤ 0.05, ** indicates *P* ≤ 0.01, *** indicates *P* ≤ 0.001)

In normal physiological conditions, ApoE is localized in cytosol. ApoE nuclear localization has been reported in ovarian cancer cells, where it leads to better survival possibly through gene regulation [47]. We treated neuro2a cells with 50 nM of HDAC6 ZnF UBP domain to observe its effect on distribution and localization of ApoE in cells. It was observed that upon HDAC6 ZnF UBP treatment, there was an increase in nuclear localization of ApoE (Fig. 6a). The localization of ApoE was quantified by analyzing the intensity of ApoE immunofluorescence in nucleus and cytoplasm of untreated and HDAC6 ZnF UBP-treated cells (No. of fields selected for quantification, n = 10 and the statistical analysis carried out by using two-tailed unpaired student t-test.). The ApoE level in cytoplasm of both untreated and HDAC6 treated cells showed no change while it was significantly increased in the nucleus of HDAC6 ZnF UBP-treated cells (Fig. 6b). The enhanced nuclear localization of ApoE may indicate an improved neuronal health [48].

### Enhanced Tubulin localization to MTOC with HDAC6 ZnF UBP treatment

HDAC6 is a known interacting partner of tubulin that regulates microtubule structure and function through deacetylation. In a previous study, it was found that HDAC6 knockdown in cells along with HDAC6 inhibitor tubacin treatment does not affect microtubule growth velocity [49]. This implies that the effects of HDAC6 that are independent of its deacetylase activity may also exist. HDAC6 ZnF UBP treatment was given to neuro2a cells to observe the effect of this domain on microtubule network. Such HDAC6 ZnF UBP treatment of neuro2a cells increased tubulin localization around nucleus in MTOC (Fig. 6c). When neuro2a cells were mapped for actin and tubulin, untreated cells showed more axonal localization of tubulin along with actin, while HDAC6 treated cells showed tubulin predominantly in MTOC (Additional file 1: Fig. 3a, b). The re-orientation of MTOC is a complex process, which occurs in a dynein, cdc42, and dynactin-dependent manner [50, 51]. Our results suggest the possible role of HDAC6 ZnF UBP domain in microtubule organization. However, the molecular mechanisms and functions of MTOC re-orientation are poorly understood.

## Discussion

Histone deactylases are known to be the enzymes that act on histones and regulates epigenetic function in nucleus. However, many non-histone substrates of HDACs in cytoplasm have been established [10]. Among the classes of histones deacetylase, class IIb deacetylase HDAC6 holds a distinctive position, being the major cytoplasmic deacetylase having two active catalytic domains [10, 52]. HDAC6 exhibit domain-wise functions, which may or may not be dependent on each other. HDAC6 plays a major role in cell proliferation, cell migration, misfolded protein degradation and stress response through its deacetylase function or interacting with various other proteins [53]. HDAC6 levels are found to be elevated in AD patients following increase in the aggregate burden in neuronal cells [15]. This suggests the possibility of involvement of this protein and its HDAC6 ZnF UBP domain in tackling aggregates in a deacetylase independent manner. Neuro2a cells treated with various concentrations of HDAC6 ZnF UBP to assess their viability upon treatment using MTT assay and LDH assay. The presence of HDAC6 ZnF UBP showed no toxicity on Neuro2a. pT181 and AT8 (pS202/pT205) are two pathological phosphorylation events of Tau implicated in AD [20]. Okadaic acid treatment increases the level of these two epitopes by inhibiting PP2a activity. Treatment of cells with HDAC6 ZnF UBP decreased the levels of both pT181 and AT8. The effect possibly arose from replenishing the PP2a activity upon inhibition by OA rather than via inhibition of Tau phosphorylation [54] Class II HDACs—HDAC1, HDAC6, and HDAC10, can form molecular complexes with phosphatases (PP1 or PP2a). However, HDAC6 was shown to interact only with the catalytic subunit of PP1 to form a complex retaining both phosphatase and deacetylase catalytic activity [55]. The binding of HDAC6 to PP1 was mapped to the second catalytic domain and C-terminal domain of HDAC6, which corresponds to ZnF UBP domain [54]. The level of GSK-3β in its phosphorylated and non-phosphorylated form determines its activity as a kinase. GSK-3β is known to associate with HDAC6 to counteract its function to induce LPS-tolerance in astrocytes [56]. GSK-3β is a versatile protein kinase with more than hundred substrates [42]. GSK-3β acts on Tau in two different manners; *i.e.*, on pre-phosphorylated primed Tau and unprimed Tau [23]. In order for GSK-3β to work on primed substrate, the N-terminal primed substrate binding domain provides binding-site for primed substrates, thereby up-regulating the kinase activity of GSK-3β. Phosphorylation of GSK-3β at Serine 9 is the regulatory mechanism to down-regulate activity of this kinase [22]. N-terminal domain with pSer9 acts as a pseudosubstrate for the primed substrate binding-domain of GSK-3β restricting the binding of other primed substrates, such as Tau [57]. Modulation of GSK-3β by HDAC6 ZnF UBP may involve regulation of Akt via PP1. PP1 dephosphorylates Akt, which is a negative regulator of GSK-3β in its phosphorylated state [58].HDAC6 ZnF UBP was found to increase the level of pSer9 phosphorylation on GSK-3β indicative of down-regulated GSK-3β activity with HDAC6 treatment in neuro2a cells.

Cellular functions, such as cell growth, morphogenesis, migration, intracellular transport, and attachment require co-ordinated operation of cytoskeletal networks of actin and microtubules. Both Tau and HDAC6 are known to be involved in the regulation of both these cytoskeletal networks [59]. Although HDAC6 exerts its regulatory function through catalytic domain, the role of HDAC6 ZnF UBP domain in this aspect needs to be explored. We studied the effects of HDAC6 ZnF UBP, on cytoskeletal organization and found the ability of this domain to restructure cytoskeletal network. HDAC6 ZnF UBP treatment resulted in enhancement of neuritic extensions. HDAC6 is an important modulator of cell migration and structure by associating with actin and tubulin polymerization machinery of cells [33, 49]. Podosomes are actin-based structures involved in the remodeling of extracellular matrix (ECM) and cell migration, while podonut consists of a cluster of podosomes interacting with ECM [60]. Podosome consists of an actin-rich core surrounded by actin regulatory molecules, such as cortactin and arp2/3, as well as cell adhesion molecules, such as Talin and Vinculin [61]. We observed the co-localization of actin and HDAC6 in neurite extensions and formation of podosomes structures involved in cell migration in HDAC6 ZnF UBP treated cells. Podosomes are membranes invaginations formed in cells like macrophages, dendritic, smooth muscle, invasive cancer cells and in post-synaptic apparatus [62]. The observed results revealed the ability of HDAC6 ZnF UBP to enhance cell migration and neurite extensions by promoting actin assembly along cell periphery. The role of HDAC6 in cytoskeletal organization has been studied extensively. HDAC6 has been reported to play regulatory role in chemotactic movement and migration of lymphocytes in deacetylase independent manner [63]. HDAC6 also regulate membrane ruffle formation in HSP90 and Rac1 mediated mechanism and found to be localized in membrane ruffles along with actin [64]. In this aspect, the role of HDAC6 ZnF UBP in promoting neuritic extensions by actin remodelling can prove to be a novel aspect of HDAC6 in neuroprotection. As previously observed, HDAC6 ZnF UBP-treated cells showed more neuritic extensions and dense actin cytoskeleton. In previous studies, Tau and actin were shown to co-localize in neuritic extensions, where they facilitated growth cone transition [65]. HDAC6 ZnF UBP treatment also leads to enhanced localization of tubulin around nucleus signifying the possible structural and pathological aspect of HDAC6 ZnF UBP. ApoE is the major apolipoprotein of central nervous system, produced primarily by astrocytes. ApoE can get translocated into nucleus through a nuclear targeting chaperone nucleolin [47]. ApoE contains a weak nuclear localizing sequence, which indicates that there must be other mechanisms involved in ApoE nuclear transport. Nucleolin is known to associate with apolipoproteins, facilitating their translocation [66]. HDAC6 may mediate ApoE translocation through HSP90 as it is known to stabilize nucleolin during mitosis [67]. However, the exact mechanism needs to be further elucidated.

**Fig. 7:**
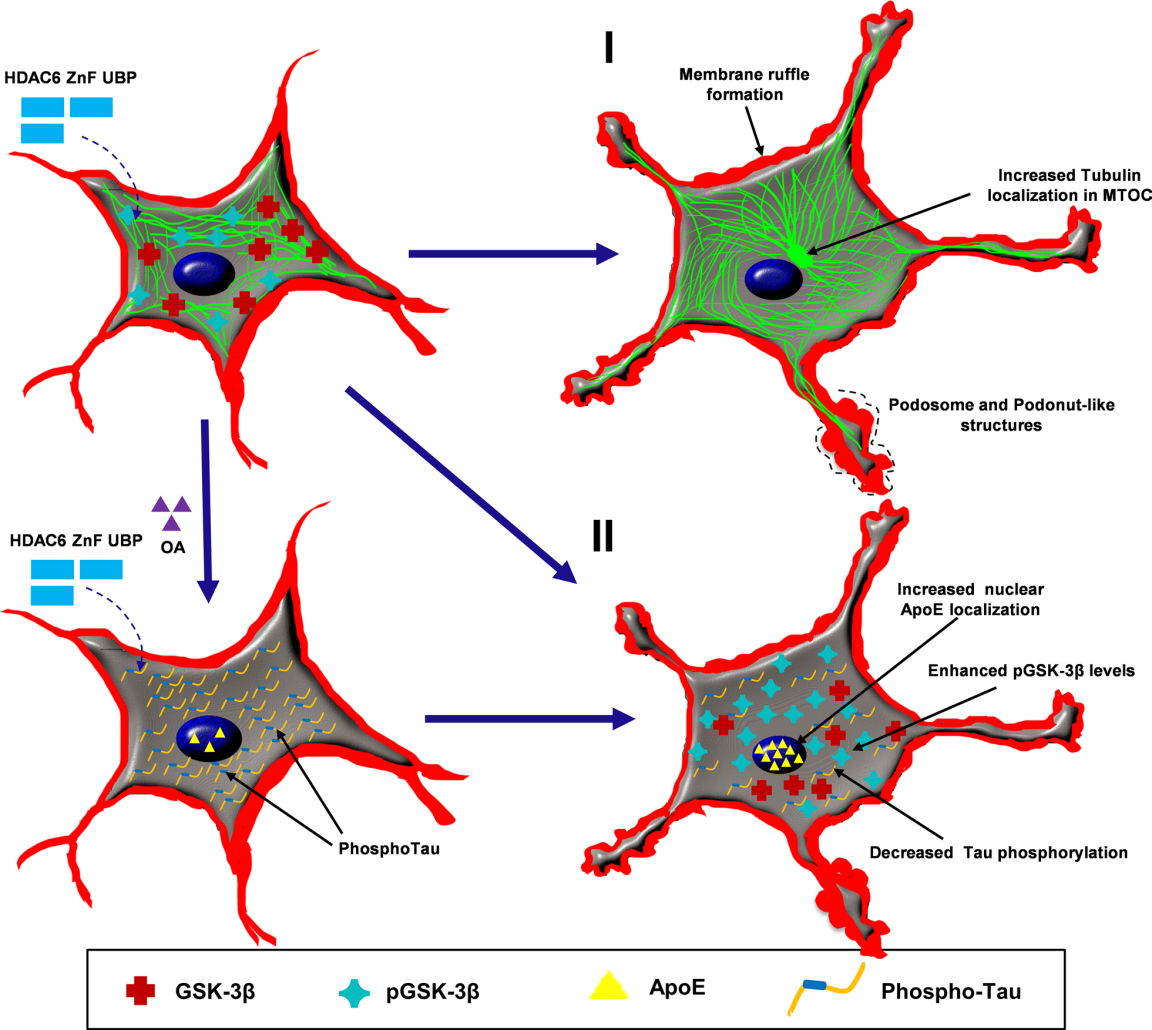
Effect of HDAC6 ZnF UBP on various cellular functions. HDAC6 ZnF UBP domain was observed to play possible roles in different aspects of neuronal cell function and morphology. Broadly classifying, we observed their effect in remodelling actin and tubulin as well as regulating Tau phosphorylation and GSK- 3β activity. (1) HDAC6 ZnF UBP treatment to neuro2a cells resulted in formation of podosome or lamellipodia-like structures and enhanced neurite extension. On the other hand, tubulin was found to be more localized in MTOC in HDAC6 treated cells while untreated cells showed uniform tubulin distribution. (2) Phosphorylation of Tau was also found to be reduced when HDAC6 ZnF UBP was given to cells along with OA as inducer of phosphorylation. OA treated cells showed increased phosphoTau levels (pT181 and AT8) while the levels of both were reduced in treated cells. The level of pGSK-3β was found to be increased with HDAC6 ZnF UBP treatment while total GSK-3β remains unaltered. Another aspect of HDAC6 treatment was observed in the localization of ApoE. Enhanced nuclear localization was found in treated cells suggesting the role of HDAC6 in regulating ApoE-mediated functions

The function of HDAC6 ZnF UBP domain is known with respect to aggregate clearance by mediating aggresome formation. However, current findings suggest that this domain may have other regulatory functions (Fig. 7). Overall, our studies suggest the role of HDAC6 ZnF UBP domain in modulating functions crucial to neuronal health that are independent of HDAC6 catalytic activity.

## Conclusions

We studied the role of HDAC6 ZnF UBP domain in different aspects of AD pathology. Upon HDAC6 ZnF UBP treatment, inactive phosphorylated form of GSK-3β increases without any change in total GSK-3β level. Decreased level of Tau phospho-epitopes pT181 and AT8 with HDAC6 ZnF UBP treatment indicates that it has a regulatory role in Tau phosphorylation. Also, HDAC6 ZnF UBP was found to be involved in cytoskeletal re-organization by modulating actin dynamics and tubulin localization. Enhanced neuritic extension and formation of podosome-like structure were observed with HDAC6 ZnF UBP treatment and tubulin was more localized in MTOC suggesting its regulatory function. ApoE translocation in nucleus enhanced upon HDAC6 ZnF UBP treatment implying its possible role in ApoE pathological cascade in AD. Overall, our study suggests that the UBP ZnF domain of HDAC6 performs various regulatory functions apart from its classical function in aggresome formation in protein misfolding diseases.

## Supplementary Information

**Additional file 1: Supplementary figure 1**. HDAC6 ZnF UBP treatment to neuro2a cells does not have a toxic effect. A) Amino acid sequence of human HDAC6 Zinc finger ubiquitin-binding domain. This domain is located on the C-terminal of HDAC6 and associates with polyubiquitinated protein aggregates to mediate the formation of aggresomes. B) MTT assay was carried out to determine the viability of neuro2a upon HDAC6 ZnF UBP treatment. Neuroblastoma cells treated with HDAC6 at different concentrations show minimum toxicity and maintain viability at 80% at highest concentration of 500 nM. C) LDH release assay determines the damage to the cell membrane upon exposure to test molecule. The membrane leakage assay (LDH assay) shows that HDAC6 does not disrupt the cell membrane and affect cell viability. D) Apoptosis rate of neuro2a cells upon HDAC6 ZnF UBP treatment was assayed using Caspase-3 assay. Caspase-3 assay shows increased levels of caspase-3 with successive time interval but do not differ from control samples. Statistical significance determined by two-tailed unpaired t-test. (n.s. – non-significant, * indicates P ≤0.05, ** indicates P ≤ 0.01, *** indicates P ≤ 0.001). **Supplementary figure 2**. Internalization of HDAC6 ZnF UBP in neuro2a. Neuro2a cells treated with HDAC6 ZnF UBP (20-500 nM) were mapped by antibody against HDAC6 and anti His-tag antibody to determine the internalization of 14 KDa HDAC6 ZnF UBP domain in cells. **Supplementary figure 3**. Co-localization of actin and tubulin in neuro2a. A) Immunostaining for β-actin and tubulin was performed to examine their localization in HDAC6 ZnF UBP treated neuro2a cells. Actin and tubulin co-localizes in the neuronal extensions in both treated and untreated cells. However, tubulin was found to be localized more in MTOC in case of HDAC6 treatment. B) Orthogonal projection images for actin and tubulin shows both actin and tubulin in untreated control cells while differential localization of tubulin in HDAC6 treated cells. **Supplementary figure 4**. DIC for neurite extensions in HDAC6 ZnF UBP treated cells. HDAC6 ZnF UBP treated cells showed enhancement in actin regulated membrane extensions in the form of podosomes, lamellipodia and podonuts. DIC images for untreated and HDAC6 treated groups indicate marked difference in the pattern of their membrane extensions.

## Data Availability

Not applicable.

## Abbreviations

AD: Alzheimer’s disease
ApoE: Apolipoprotein E
ECM: Extracellular matrix
FTDP-17: Frontotemporal dementia and parkinsonism linked to chromosome 17
GSK-3β: Glycogen synthase kinase-3beta
HDAC6: Histone deacetylase 6
HDAC6 ZnF UBP: HDAC6 zinc finger ubiquitin binding protein domain
HSP90: Heat shock protein 90
IDP: Intrinsically disorder protein
IDPR: Intrinsically disorder protein region
MTOC: Microtubule organizing centre
NFTs: Neurofibrillary tangles
OA: Okadaic acid
PP1: Protein phosphatase 1
PP2A: Protein phosphatase 2A
UPS: Ubiquitin proteasomal system
