# Supplementary material for: The extracellular HDAC6 ZnF UBP domain modulates the actin network and post-translational modifications of Tau
Source: Cell Commun Signal. 2021 May 1;19:49. doi: 10.1186/s12964-021-00736-9 (PMC8088071; doi:10.1186/s12964-021-00736-9)
Supplement: Supplementary file 2 — Additional file 1: Supplementary figure 1. HDAC6 ZnF UBP treatment to neuro2a cells does not have a toxic effect. A) Amino acid sequence of human HDAC6 Zinc finger ubiquitin-binding domain. This domain is located on the C-terminal of HDAC6 and associates with polyubiquitinated protein aggregates to mediate the formation of aggresomes. B) MTT assay was carried out to determine the viability of neuro2a upon HDAC6 ZnF UBP treatment. Neuroblastoma cells treated with HDAC6 at different concentrations show minimum toxicity and maintain viability at 80% at highest concentration of 500 nM. C) LDH release assay determines the damage to the cell membrane upon exposure to test molecule. The membrane leakage assay (LDH assay) shows that HDAC6 does not disrupt the cell membrane and affect cell viability. D) Apoptosis rate of neuro2a cells upon HDAC6 ZnF UBP treatment was assayed using Caspase-3 assay. Caspase-3 assay shows increased levels of caspase-3 with successive time interval but do not differ from control samples. Statistical significance determined by two-tailed unpaired t-test. (n.s. – non-significant, * indicates P ≤0.05, ** indicates P ≤ 0.01, *** indicates P ≤ 0.001). Supplementary figure 2. Internalization of HDAC6 ZnF UBP in neuro2a. Neuro2a cells treated with HDAC6 ZnF UBP (20-500 nM) were mapped by antibody against HDAC6 and anti His-tag antibody to determine the internalization of 14 KDa HDAC6 ZnF UBP domain in cells. Supplementary figure 3. Co-localization of actin and tubulin in neuro2a. A) Immunostaining for β-actin and tubulin was performed to examine their localization in HDAC6 ZnF UBP treated neuro2a cells. Actin and tubulin co-localizes in the neuronal extensions in both treated and untreated cells. However, tubulin was found to be localized more in MTOC in case of HDAC6 treatment. B) Orthogonal projection images for actin and tubulin shows both actin and tubulin in untreated control cells while differential localization of tubulin in HDAC6 treated cells. [file 12964_2021_736_MOESM2_ESM.pdf]

Supplementary information

The extracellular HDAC6 ZnF UBP domain modulates the actin network and posttranslational modifications of Tau

Abhishek Ankur Balmik[1,2,#], **Shweta Kishor Sonawane**[1,2,#] **and Subashchandrabose Chinnathambi**[1,2,§]

[1]Neurobiology Group, Division of Biochemical Sciences, CSIR-National Chemical Laboratory, Dr. Homi Bhabha Road, 411008 Pune, India

[2]Academy of Scientific and Innovative Research (AcSIR), Ghaziabad, 201 002, India

[#] These authors have equally contributed.

[§]To whom correspondence should be addressed**: Prof. Subashchandrabose Chinnathambi**, Neurobiology group, Division of Biochemical Sciences, CSIR-National Chemical Laboratory (CSIR-NCL), Dr. Homi Bhabha Road, 411008 Pune, India, Telephone: +91-20-25902232, Fax. +91-20-25902648. **Email:** s.chinnathambi@ncl.res.in

Additional file 1

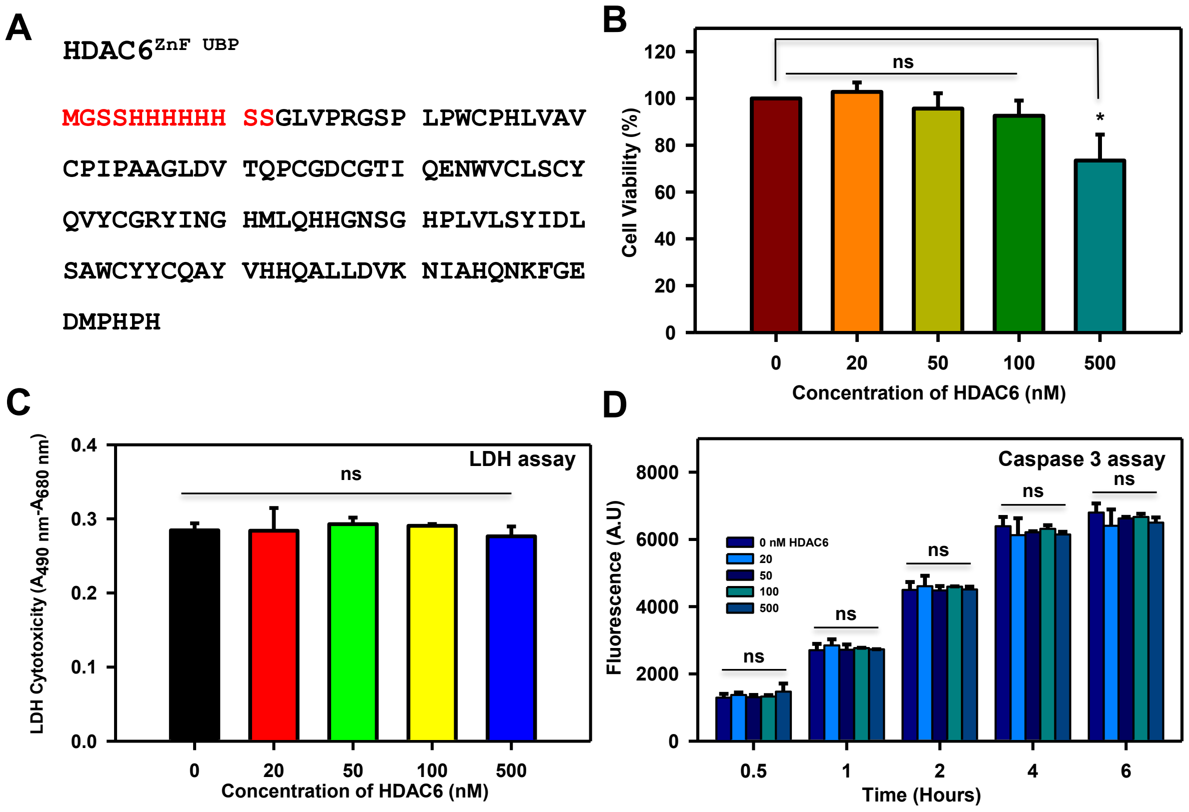

Additional file 1. Supplementary figure 1. HDAC6 ZnF UBP treatment to neuro2a cells does not have a toxic effect. A) Amino acid sequence of human HDAC6 Zinc finger ubiquitin-binding domain. This domain is located on the C-terminal of HDAC6 and associates with polyubiquitinated protein aggregates to mediate the formation of aggresomes. B) MTT assay was carried out to determine the viability of neuro2a upon HDAC6 ZnF UBP treatment. Neuroblastoma cells treated with HDAC6 at different concentrations show minimum toxicity and maintain viability at 80% at highest concentration of 500 nM. C) LDH release assay determines the damage to the cell membrane upon exposure to test molecule. The membrane leakage assay (LDH assay) shows that HDAC6 does not disrupt the cell membrane and affect cell viability. D) Apoptosis rate of neuro2a cells upon HDAC6 ZnF UBP treatment was assayed using Caspase-3 assay. Caspase-3 assay shows increased levels of caspase-3 with successive time interval but do not differ from control samples. Statistical significance determined by two-tailed unpaired t-test. (n.s. – non-significant, * indicates $P \leq 0.05$, ** indicates $P \leq 0.01$, *** indicates $P \leq 0.001$).

Additional file S2

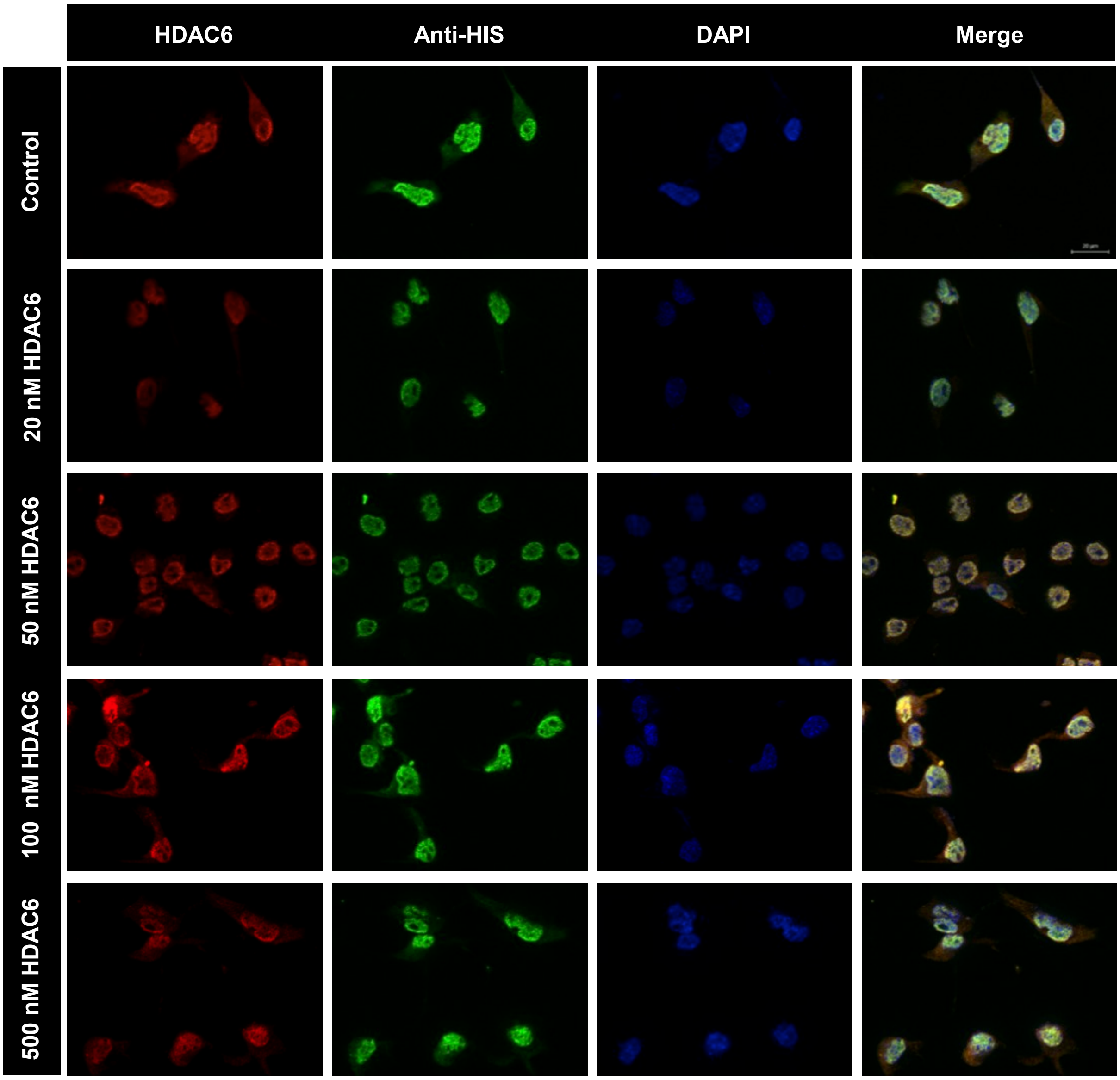

Additional file 2. Supplementary figure 2. Internalization of HDAC6 ZnF UBP in neuro2a. Neuro2a cells treated with HDAC6 ZnF UBP (20-500 nM) were mapped by antibody against HDAC6 and anti His-tag antibody to determine the internalization of 14 KDa HDAC6 ZnF UBP domain in cells.

Additional file S3

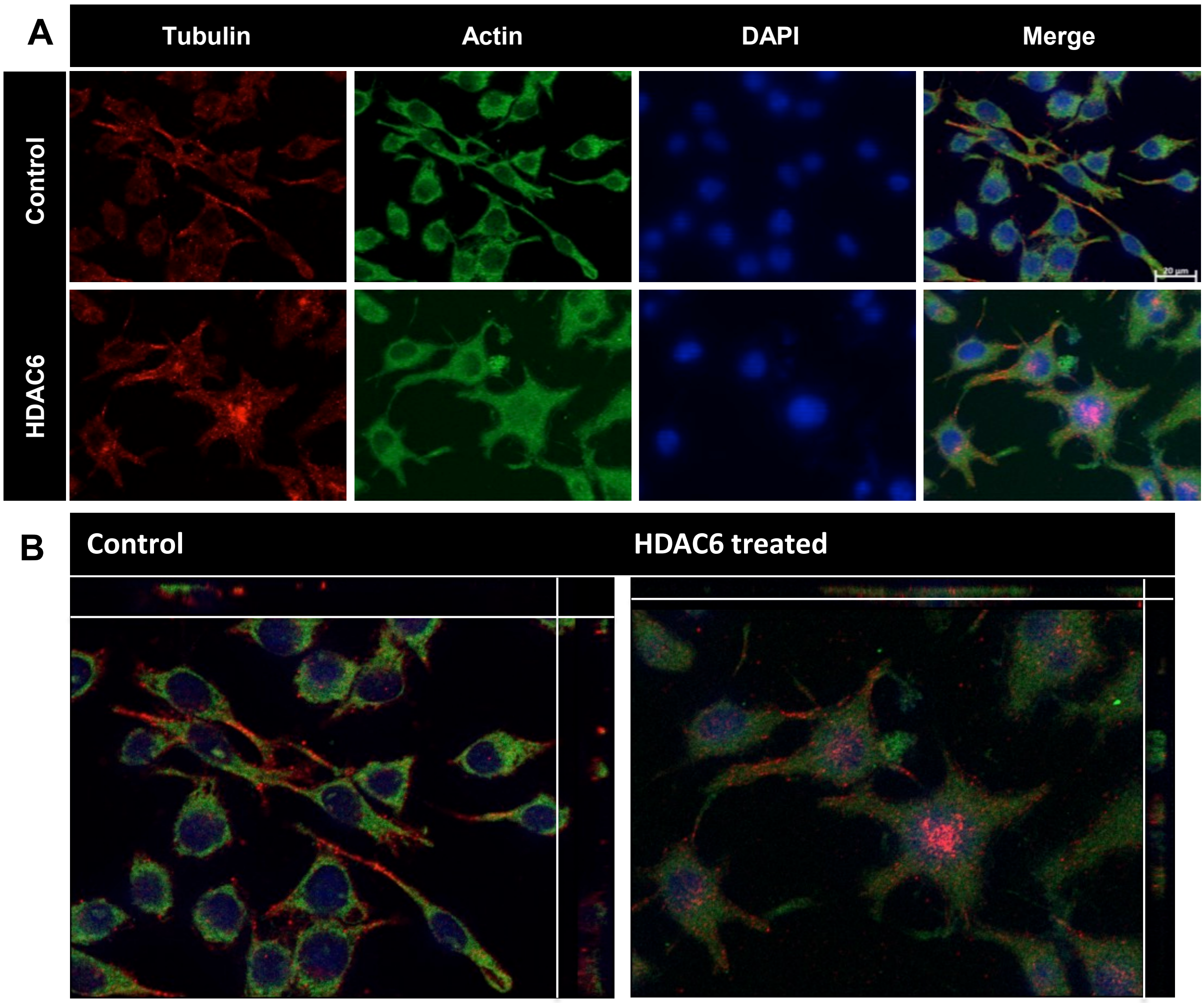

Additional file 3. Supplementary figure 3. Co-localization of actin and tubulin in neuro2a. A) Immunostaining for β-actin and tubulin was performed to examine their localization in HDAC6 ZnF UBP treated neuro2a cells. Actin and tubulin co-localizes in the neuronal extensions in both treated and untreated cells. However, tubulin was found to be localized more in MTOC in case of HDAC6 treatment. B) Orthogonal projection images for actin and tubulin shows both actin and tubulin in untreated control cells while differential localization of tubulin in HDAC6 treated cells.

Additional file S4

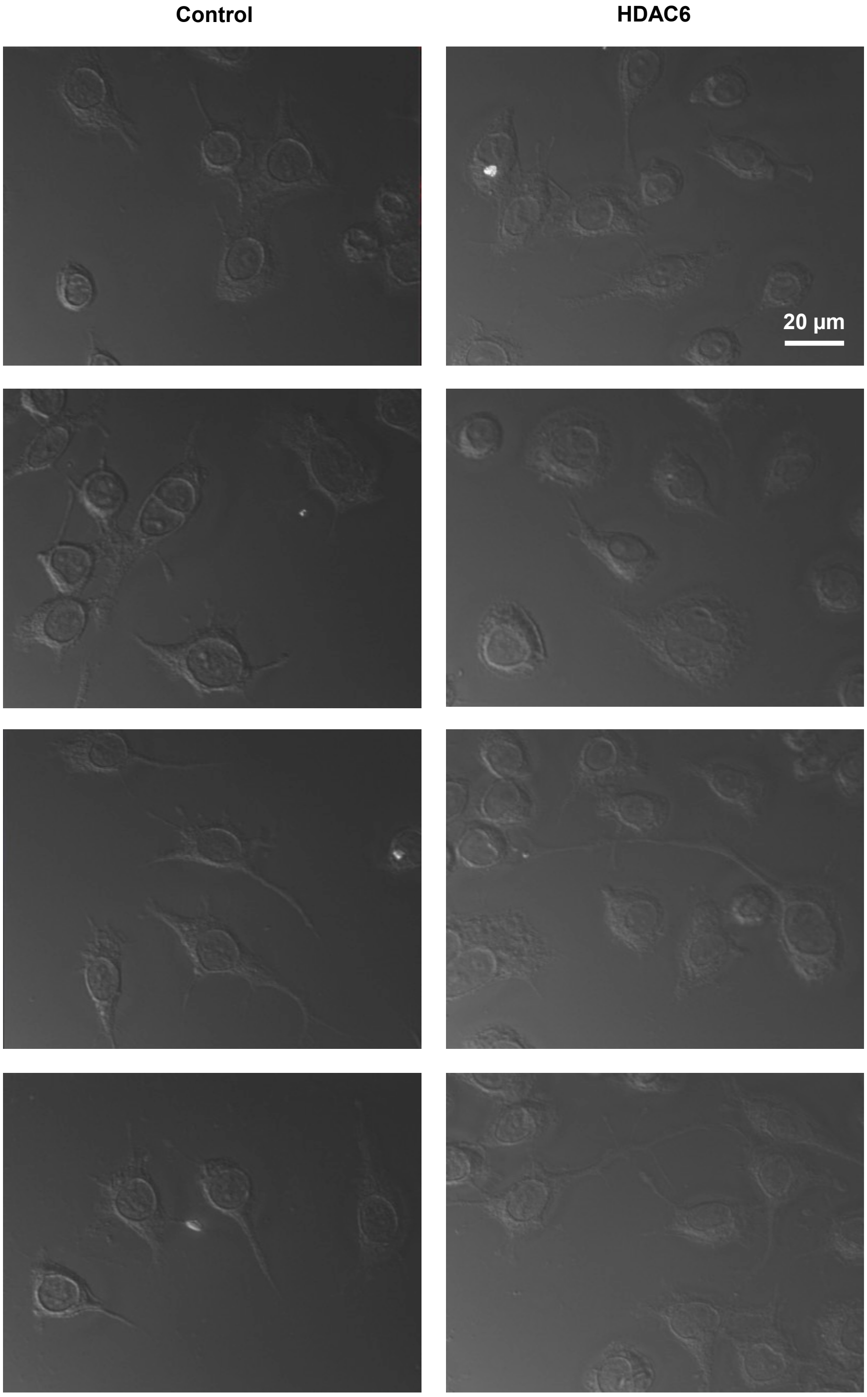

Additional file 4. Supplementary figure 4. DIC for neurite extensions in HDAC6 ZnF UBP treated cells. HDAC6 ZnF UBP treated cells showed enhancement in actin regulated membrane extensions in the form of podosomes, lamellipodia and podonuts. DIC images for untreated and HDAC6 treated groups indicate marked difference in the pattern of their membrane extensions.
